# Poker-DVS and MNIST-DVS. Their History, How They Were Made, and Other Details

**DOI:** 10.3389/fnins.2015.00481

**Published:** 2015-12-22

**Authors:** Teresa Serrano-Gotarredona, Bernabé Linares-Barranco

**Affiliations:** Instituto de Microelectrónica de Sevilla (IMSE-CNM), CSIC and Universidad de SevillaSevilla, Spain

**Keywords:** event-driven vision, event-driven object recognition, dynamic vision sensor (DVS), address event representation, high speed vision, frame-free vision

## Abstract

This article reports on two databases for event-driven object recognition using a Dynamic Vision Sensor (DVS). The first, which we call Poker-DVS and is being released together with this article, was obtained by browsing specially made poker card decks in front of a DVS camera for 2–4 s. Each card appeared on the screen for about 20–30 ms. The poker pips were tracked and isolated off-line to constitute the 131-recording Poker-DVS database. The second database, which we call MNIST-DVS and which was released in December 2013, consists of a set of 30,000 DVS camera recordings obtained by displaying 10,000 moving symbols from the standard MNIST 70,000-picture database on an LCD monitor for about 2–3 s each. Each of the 10,000 symbols was displayed at three different scales, so that event-driven object recognition algorithms could easily be tested for different object sizes. This article tells the story behind both databases, covering, among other aspects, details of how they work and the reasons for their creation. We provide not only the databases with corresponding scripts, but also the scripts and data used to generate the figures shown in this article (as Supplementary Material).

## 1. Introduction

The availability of good, challenging benchmarks is an important prerequisite when developing and comparing algorithms and tools. In the field of traditional frame-based vision, machine vision researchers have made extensive use of a number of well established benchmark problems (such as MNIST, LeCun et al., 1998 and Caltech101, Fei-Fei et al., 2007). More recently, Frame-Free Event-Driven Vision Sensors (Culurciello et al., 2003; Chen and Bermak, 2005; Costas-Santos et al., 2007; Leñero-Bardallo et al., 2010, 2014), and, above all, devices known as “Dynamic Vision Sensors” (DVS) (Lichtsteiner et al., 2008; Leñero-Bardallo et al., 2011; Posch et al., 2011; Serrano-Gotarredona and Linares-Barranco, 2013a; Brandli et al., 2014; Yang et al., 2015) which allow for higher pixel resolution, have also been used by some research groups for vision related applications. To date, however, not many groups yet have access to this new sensor technology and are able to use it to record scenes to test the event-driven algorithms and architectures they are developing. Furthermore, even if these new sensors were readily available and accessible to everybody, it would be highly convenient to have benchmark problems with which independent researchers could test their event-driven vision algorithms and systems on the same benchmark databases for more objective comparisons. By their nature, datasets recorded with DVS cameras pose an extra challenge with respect to traditional Frame-based image benchmarks: since a DVS sensor is sensitive only to changes, the scenes must be moving. One can move either the scene itself or the sensor, to capture edges and textures of objects, but it must be remembered that in both cases the datasets recorded with DVS cameras will be moving, and this is problematic for event-driven object recognition systems. In a first stage, it is possible to create the “illusion” that an object is static by subtracting the “Center of Mass” coordinate of the object of interest. However, in the long term, recognition algorithms and systems need to feature simultaneous tracking and recognition. This article, submitted to the Frontiers Special Issue on “*Benchmarks and Challenges for Neuromorphic Engineering*,” discusses two datasets developed in our group in the past. The first, called Poker-DVS, was never intended to be used as a public benchmark, but was conceived just to illustrate a very high speed object recognition problem with event-driven convolutional neural networks (Pérez-Carrasco et al., 2013). In its original form, it consisted of just 40 poker pips appearing on the screen for about 20–30 ms. The pips were artificially made static by tracking and subtracting their moving “Center of Mass.” We have now extended those same original recordings to create a set of 131 elements. The second dataset, which we call MNIST-DVS, was announced in several mailing lists in December 2013 and made available on the internet (Serrano-Gotarredona and Linares-Barranco, 2013b). Since then, it has been used in a small number of studies carried out by researchers like (Henderson et al., 2015; Zhao et al., 2015). In this article we explain the history and motivation of both datasets, focusing on certain intricate peculiarities which were never officially reported and other aspects of interest.

## 2. The Poker-DVS dataset

The history of Poker-DVS can be traced back to the CAVIAR project (Serrano-Gotarredona et al., 2009), for which our group developed event-driven convolution chips and Tobi Delbrück's group developed the Dynamic Vision Sensor (DVS; Lichtsteiner et al., 2008). After that project, although we then had a sample DVS to experiment with, Teresa Serrano-Gotarredona decided that our group should design and fabricate our own DVS camera, since devices of this type were not commercially available and were a key element in event-driven vision research. The original idea was simply to redesign Delbrück's DVS with a faster event read-out scheme (Boahen, 2000), but during the pixel design phase an extra gain stage was added to improve contrast sensitivity as well. When Teresa took maternity leave, our PhD student Leñero took charge of testing the first prototype (Leñero-Bardallo et al., 2011). The design and test results were included in his PhD Dissertation, along with another event-driven spatial contrast vision sensor he had developed and tested (Leñero-Bardallo et al., 2010). In 2010 Tobi Delbrück was invited, as an obvious choice, to Leñero's PhD defense, which included a live demonstration of the new DVS sensor. Those of us who know Tobi Delbrück personally are aware that he is a big fan of poker. He usually carries a deck of cards around with him and does tricks with it when relaxing at meal times especially if there are children around. He also organizes “Poker Night” at each year's Telluride Neuromorphic Engineering Workshop. After Leñero's defense, Delbrück went to the demo setup, took a card deck from his pocket and quickly browsed it in front of the DVS sensor. He then asked Leñero to play the recording back in slow motion. He seemed quite impressed to find that the DVS camera was able to capture all the details on the cards correctly, given the very high speed of its event rate (close to 8 Meps—mega events per second). From then on, we continued to use this poker card browsing demo in conferences and lectures to illustrate the good performance both of this DVS sensor and of an improved version with much lower power and a smaller pixel area (Serrano-Gotarredona and Linares-Barranco, 2013a). After some practice, we managed to browse a full deck of 52 cards in just 0.65 s, producing a total of 0.5 million events with a peak rate of slightly above 8 Meps (Camuñas-Mesa et al., 2012).

At that time we were collaborating with the Signal Processing Department at the University of Seville exploring event-driven processing architectures and systems mainly through simulations. It was there that the AERST event-driven simulator was first developed (Pérez-Carrasco et al., 2010) and applied to a texture recognition scenario. But since we also wanted to extend this work to event-driven object recognition we used the DVS to record a few scenes of people walking. The recordings were rotated through different angles (0, 90, 180, 270) and we decided to try to create a recognition system capable of detecting the angle of rotation. To this end we developed an event-driven convolutional neural network system, and then trained it and tested it with the rotated silhouette recordings. The work was submitted to IEEE Transactions on Pattern Analysis and Machine Intelligence, but was rejected with the comment that the results were not very impressive. At this point, Bo Zhao, a PhD student from Prof. Chen's laboratory in Singapore, arrived on a 5-month visit to our Institute. He wanted to familiarize himself with DVS data and event-driven processing, and we thought it would be an interesting exercise for him to retrain the rotating human silhouettes ConvNet with high speed poker pips crossing the screen. This would produce a very high speed event-driven recognition system and, hopefully, impress the reviewers of our rejected manuscript more than our previous submission.

The first thing we noticed at this point was that the DVS poker card recordings we had made so far were not good enough for reliable recognition. Browsing a poker card deck in front of a DVS camera produces a very high event traffic rate. Furthermore, our DVS cameras (Leñero-Bardallo et al., 2011; Serrano-Gotarredona and Linares-Barranco, 2013a) had about 10 times higher contrast sensitivity than the DVS camera first reported by Delbrück and would therefore produce extremely high traffic (and actually saturate the internal event arbiter)^1^ if contrast was set at maximum sensitivity. Consequently, to record high speed poker card browsing (i.e., the full deck in 1–3 s), the DVS contrast sensitivity had to be set rather low, so that the peak event rate stayed within the 8–10 Meps range. Another major speed bottleneck was the system connected to the DVS output port. In our case we had two options. We could use the USBAER board or the USBAERmini2 board, both of which had been developed during the CAVIAR project (Serrano-Gotarredona et al., 2009). The first board supports a fully standalone mode (communicating only with the sensor at maximum speed). When loaded with the data-logger firmware it can read events up to a rate of about 9 Meps. Unfortunately, however, it can only read 0.5 Mega events, which is the limit of its on-board storage capacity. This half mega event can later be downloaded to a computer via a low-speed USB. With the USBAERmini2 board the maximum event rate is strongly influenced by the host computer, since events are directly transferred in real time from the board to the PC via high-speed USB2.0. Our experience is that with a top-of-the-range Windows PC, the maximum achievable event recording rate is less than 7 Meps. We used the USBAERmini2 with the jAER (Delbrück, 2006) open source control software provided by Tobi Delbrück's group, mainly because of this software's user-friendliness and interactivity.

**Figure 1:**
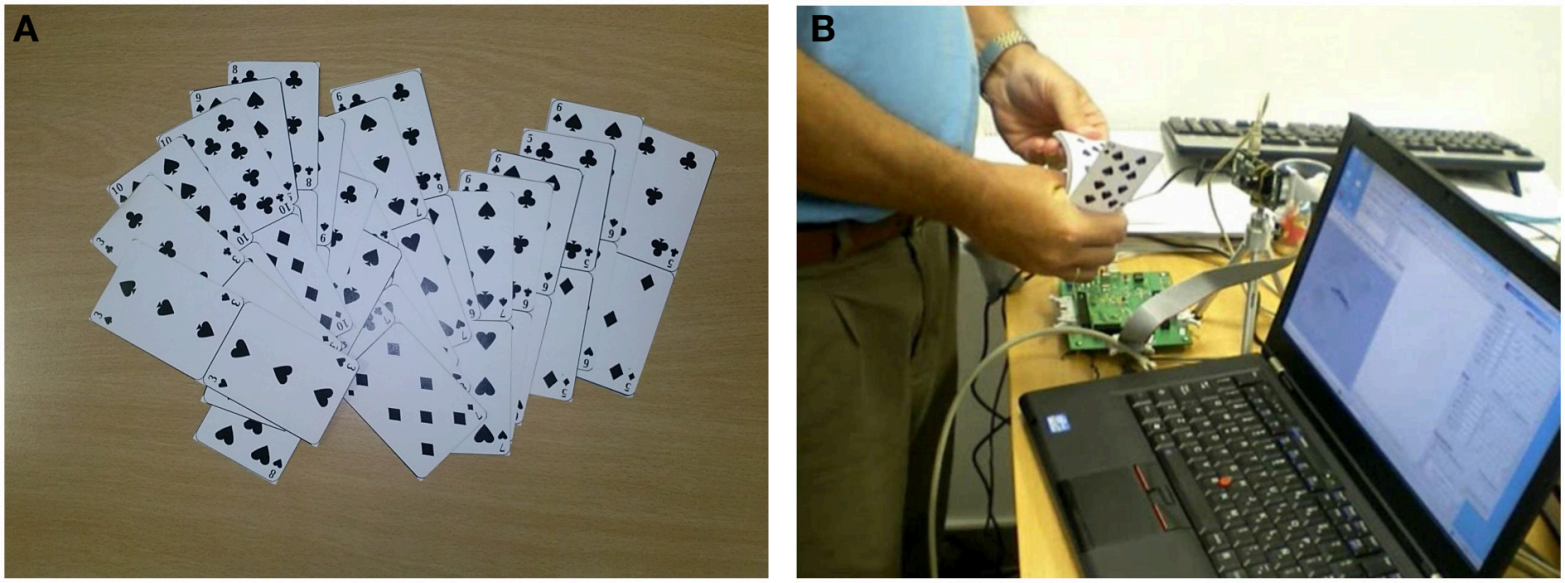
**(A)** Custom made poker card deck with all pips in black. **(B)** Browsing the custom poker card deck in front of our DVS camera.

But here came our next problem. After adjusting contrast sensitivity to a level low enough not to saturate the event read out chain (or the DVS chip internal event arbiter, or the USBAERmini2 read out board), we noticed that the red colored poker pips (hearts and diamonds) were of rather low visual quality, while the black ones (spades and clubs) were of excellent quality. We therefore decided to print out our own poker deck with all the pips in black. This way, the recordings would be of equal quality for all four suits, resulting in a good, fair dataset for this four-symbol recognition target. Figure 1A shows a photograph of our custom made card deck. Note that we cut the cards in groups of two, with pairs sharing the short edge. This way, it was possible to hold the lower part firmly with one hand, while easily browsing the upper half, which would then be better exposed to the camera, as shown in Figure 1B.

**Figure 2:**
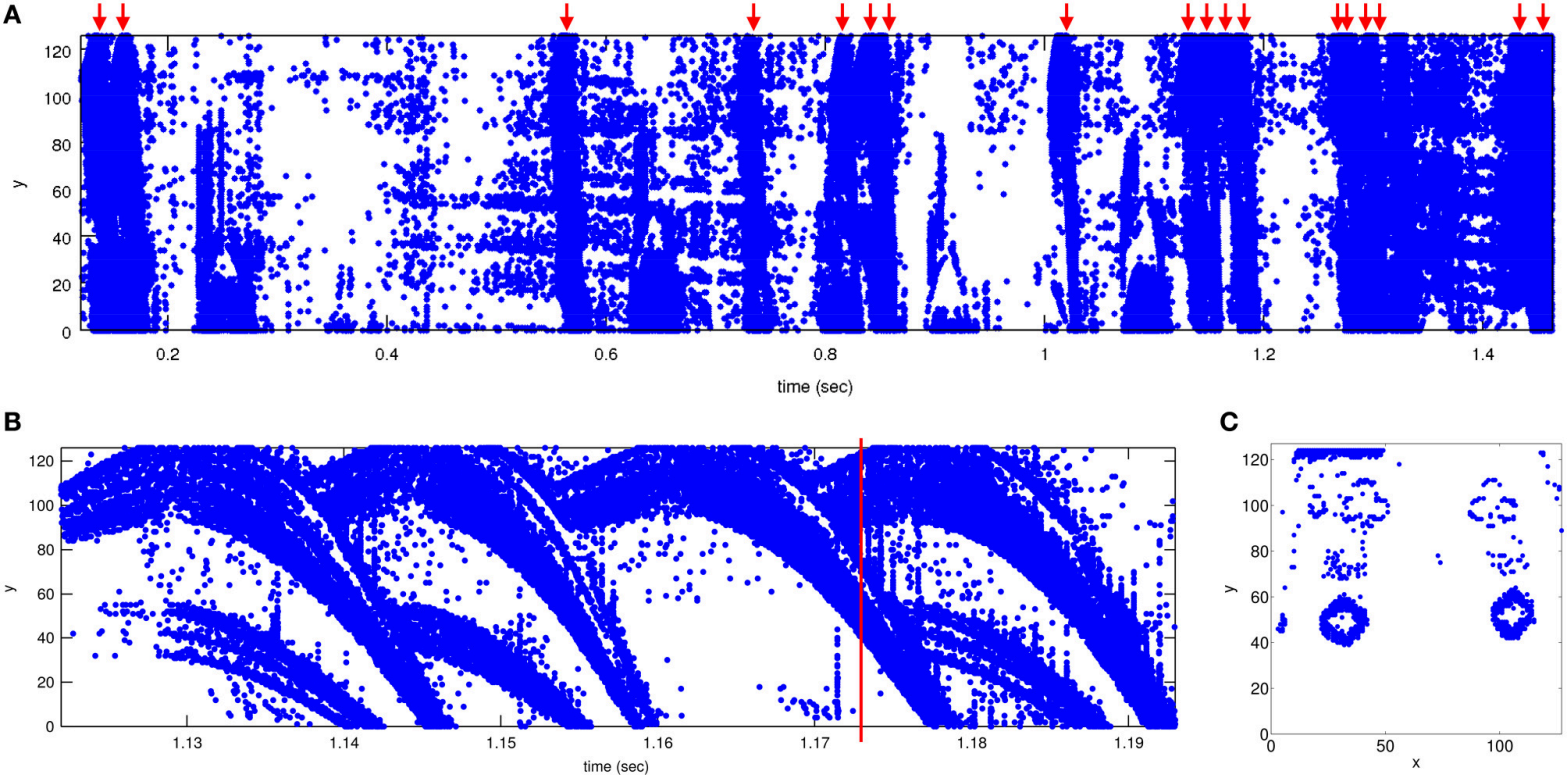
**Recorded events**. Units for *x* and *y* are pixel coordinates (from 0 to 127) and *time* is in seconds. **(A)** Events recorded when browsing 18 cards, shown in *y* vs. *time* plane. Upper red arrows indicate the position of a card in time. Total time is 1.34 s. **(B)** Zoom-in between times 1.122 and 1.193 s showing 4 sequential cards. The 4 cards cross the 128 × 128 pixel screen in 24, 23, 26, and 23 ms, respectively. **(C)** Events collected between 300 μs before and after the time mark in **(B)** displayed as *y* vs. *x*. Note how the previous card (diamonds) disappears at the bottom and the next card (clubs) appears at the top. Matlab scripts to reproduce these figures are included in the Supplementary Material.

The *y* vs. *time* plane in Figure 2A shows the events recorded while browsing 18 cards in 1.47 s, a total of 487 k events, with a peak event rate of 6 Meps. Figure 2B shows a close-up a 4 card sequence browsed in 70 ms. Note how the symbols first ramp up a little and then, after reaching the top, run down through the screen. Figure 2C shows a 600 μs capture of an instant where two cards can be seen simultaneously. Depending on a pip's position on the card, its trajectory duration can vary from a maximum of about 30 ms (for pips at the top of the cards) down to a minimum of about 5 ms (for pips at the bottom part of the cards).

After transferring these recordings to Matlab for off-line processing, we tracked 40 pips (10 for each type) as they crossed the screen and then cropped and centered them into a 31 × 31 pixel window. These post-processed recordings constituted the original dataset used to train the Event-Driven ConvNet. Bo Zhao successfully trained the network and obtained impressively high speeds in recognition performance, reducing recognition time to 1 ms after stimulus on set (in simulation). That is to say, although the pips take from 10 to 30 ms to cross the screen, it is possible to recognize them within the first 1–2 ms if they are clearly visible. Note that if you look carefully at the pip recordings with jAER, the symbols are not always clearly visible during the full sweeping period, especially at the start and end of the event sequence. These results were added to our previously rejected manuscript, which was then resubmitted, accepted and published (Pérez-Carrasco et al., 2013). The 40 pip recordings used in this paper were never made publicly available, although some researchers requested them off-line and we provided them. For this Frontiers Special Issue on Benchmarking we decided to complement that set with more pips extracted from the same recordings. We originally used 3 different custom card deck recordings, each with a small number of cards (around 18), and with different ratios of pips. From these three original card recordings we extracted a set of 131 tracked and centered pips, which now constitute the new Poker-DVS dataset (Serrano-Gotarredona and Linares-Barranco, 2015).

It should be mentioned that, although we used an automated script to track, center, and extract the 31 × 31 pixel pips, we had to identify both the starting (*x, y*) coordinate and the start and end timestamps of the pip sequences crossing the screen manually in order to extract the symbols successfully. This was because the original recordings had a significant amount of noise and other distractors, like the edges of the cards, or the card numbers in the corners. The automated tracking algorithm would often jump to track this card number, or jump to an adjacent symbol if few events were produced by the symbol of interest during a short transient. We believe that automatic tracking, recognition and extraction of the different pips from the full card browsings recorded initially would provide a very interesting, highly challenging benchmark, and therefore, together with our tracked and extracted 31 × 31 pixel pips, we are now providing the set of three original full card browsing recordings. We also present the Matlab scripts for extracting the individual 31 × 31 pixel pips from those recordings. For each pip extracted, the Matlab scripts include its initial coordinate and its initial and end timestamps, which were very patiently identified manually for each pip using jAER. Furthermore, each extracted pip sequence was carefully monitored with jAER, together with its center of mass trajectory. If the extracted sequence did not show up clearly in jAER or if there were suspicious jumps in the center of mass trajectory, given parameters (such as the radius of the extracted symbol, deviation between center of mass and actual center for asymmetric symbols, initial coordinates or initial and end timestamps) were manually retuned to improve the sequence.

### 2.1. Example uses of Poker-DVS and results

The original 40-pip Poker-DVS dataset was used on a purely feed-forward spiking ConvNet (Pérez-Carrasco et al., 2013), achieving around 90–91% recognition success rate on the training set (the data was not separated into training and test sets). Recognition could be achieved with just the first front of events, corresponding to latencies of around 1–3 ms.

Orchard et al. (2015b) subsequently proposed a very effective event-driven ConvNet architecture called H-First, which exploits lateral inhibition within each layer, testing this architecture with the original 40-pip Poker-DVS dataset, and splitting it up into a training set and a test set. They achieved recognition accuracy of 97.5% on the test set.

Recently, the original 40-pip Poker-DVS dataset has also been used by Benosman and Lagorce (Lagorce, 2015) in a newly proposed event-driven computing architecture based on space-time surfaces. These researchers integrated over the full period of card pip presentations (10–30 ms), and explored their architectures using different distance metrics. For one of the distances they tested, called “normalized distance,” they obtained 100% recognition success over the training set (they did not separate data into training and test sets).

## 3. The MNIST-DVS dataset

The Poker-DVS dataset is an extremely high-speed set, with shape changing symbols, sometimes highly distorted, sometimes fully disappearing, and usually with a lot of extra noise. These characteristics also make it a highly challenging one. We wanted to provide an alternative one, with cleaner characters, slower speed and in which the characters would last for a few seconds (2–3). With this in mind, the Frame-based MNIST dataset (LeCun et al., 1998) seemed to be a sensible choice as a reference, since it is a well established benchmark in conventional frame-based machine vision. The MNIST set consists of a huge number (70,000) of 28 × 28 pixel pictures of handwritten digits. We wanted to transform these characters into event-driven movies with the digits moving in front of a DVS camera. We also wanted to provide different scales for the digit sizes, as this would better simulate a natural environment where, for example, robots with event-driven machine vision need to be capable of detecting and recognizing objects independently of their size. Since DVS cameras are sensitive to changes, there are two ways of transforming digit pictures into events using a DVS. One is to flash the digit on a screen, either once or by (randomly) flickering the pixels. This, however, is not a very natural scenario. A robot moving in an environment, for example, would not typically be exposed to light flashing objects. We, therefore, decided to opt for the second, more difficult choice: that of moving the digits. Obviously, the best solution would have been to move digits mechanically printed on paper. This would have been the scenario most similar to the real world, but was totally impractical considering the many thousands of digits to be recorded. In our project, we decided to use an LCD monitor to display the moving characters. Monitors refresh full images at a constant rate, so we knew this method would introduce some artificial artifacts. We expected to see bursts of events at every new frame with silent periods in the last part of each frame time, so to minimize these expected artifacts we first set our high definition LCD monitor to its maximum possible contrast. This way, pixels would not be periodically switched off during a fraction of the frame time. Secondly, we set the monitor's frame frequency to its maximum possible value, which in our case was 75 Hz. Thirdly, we decided to move the digits very slowly over the screen using relatively short trajectories. This way, only a few “randomly” placed pixels would be refreshed between consecutive frames. Finally, since our DVS camera had a relatively low resolution (128 × 128 pixels) whereas our LCD monitor was high definition, we set the DVS camera to cover the maximum possible field of view on the monitor. This way, several monitor pixels would “map” to each DVS pixel, improving smoothing between the moving pixels on the monitor and those recorded with the DVS. Despite all these precautions, we never actually thought we would fully neutralize the frame refresh rate artifacts: our plan was simply to readjust the timings of the recorded events artificially (while retaining their order) to remove the effect of the LCD monitor refreshes. In our opinion, readjusting the timing, while keeping events in the same order would almost certainly have no effect on any recognition algorithm. This assumption was based on claims in many earlier works on computational neuroscience that the really meaningful representation for event computation is the rank ordering of events (once all specific time representations have been removed; Van Rullen and Thorpe, 2001), or at least that what really matters is the approximate relative timing of events and orders rather than precise absolute times. This, therefore, was our original plan.

**Figure 3:**
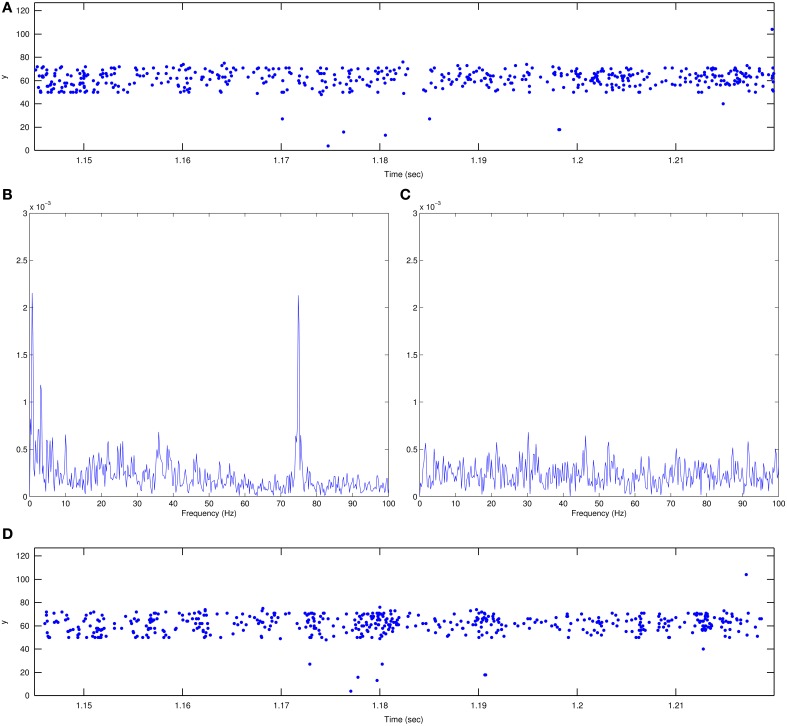
**Illustration of LCD screen refresh rate impact. (A)** Plot of recorded events in *y* vs *time* plane for a 70 ms time interval. Units for *y* are pixel number. This sequence includes five 75 Hz screen refreshes in a period of 13.33 ms. There is a slight tendency toward event grouping every 13.33 ms. **(B)** Timestamp sequence spectrum of the recorded event-driven video showing a clear peak at 75 Hz. Mean interspike time difference is 140 μs and standard deviation is 169 μs. **(C)** Spectrum of the same recorded sequence after readjusting the timestamps to remove the 75 Hz peak. Mean interspike time difference and standard deviation are kept the same as in **(B)**, while timestamps are generated randomly. **(D)** Plot of the event sequence in **(A)** after timestamp adjustment. There is still a tendency toward event grouping but its periodicity is random. Matlab scripts to reproduce these figures are included in the Supplementary Material.

However, when we started recording and looked at the resulting event sequences, we were quite surprised to see that the impact of LCD screen refresh was extremely small. Figure 3A shows a close-up of a *y* vs. *time* plot corresponding to the timing of five 75 Hz frames (about 70 ms). Although there is clearly a tendency for the events to concentrate more in 5 regions separated by 13.33 ms, the event flow is smooth overall. If the recorded sequence is carefully observed with jAER in slow motion, at different play back speeds, no appreciable artifact attributable to the LCD monitor can be seen. In the end, therefore, we decided to present the recorded sequences as they were. In any case, together with the dataset we also provide now a Matlab script for reshuffling the timings of the events, while preserving their order, as explained in the next Section.

To clearly see the impact of the 75 Hz LCD screen refresh, the only option is to perform a spectral analysis on the timestamp sequence of a recording (Orchard et al., 2015a). Figure 3B shows the analysis of one such recorded sequence (for character “0,” the smallest scale, the first recording in the dataset). There is a clear peak at 75 Hz coming out of the noise floor. This artifact can be removed by readjusting the event timings in the following manner, but without changing the order of the recorded events. First, the inter-spike time differences Δ*t*_*orig*_*i*__ = *t*_*orig*_*i*+1__ − *t*_*orig*_*i*__, mean $\bar{\Delta t}$ and standard deviation σ(Δ*t*) of the recorded events have to be computed from their timestamp vector {*t*_*orig*_1__, *t*_*orig*_2__, …*t*_*orig*_*T*__}. Then a completely new timestamp vector is generated {*t*_*new*_1__, *t*_*new*_2__, …*t*_*new*_*T*__} with the same inter-spike difference mean and standard deviation:

(1) $$t_{new_{i+1}}=t_{new_{i}}+\bar{\Delta t}+2\times \sigma (\Delta t)\times x_{i}$$

where *x*_*i*_ is a randomly generated number with normal distribution, zero mean and unit standard deviation. This new sequence has to be sorted for monotonically increasing times, and then rescaled for the same initial and end timestamps. The resulting re-timed sequence has the spectrum shown in Figure 3C, where the 75 Hz peak has been removed. When playing back both sequences in jAER no difference can be observed between the two recordings. We therefore strongly doubt that the 75 Hz artifact will have any impact on any recognition algorithm. In any case, together with the MNIST-DVS dataset we provide a Matlab script for changing the timings of all the recorded events to remove the 75 Hz artifact. This way the dataset can be tested with and without the 75 Hz artifact. As a point of interest, Figure 3D shows the same 70 ms sub-sequence as in (a) after re-timing of the timestamps. Again, there is a clear, even stronger, tendency for events to group together but the periodicity of this grouping is random and thus does not show up on the spectrum.

**Figure 4:**
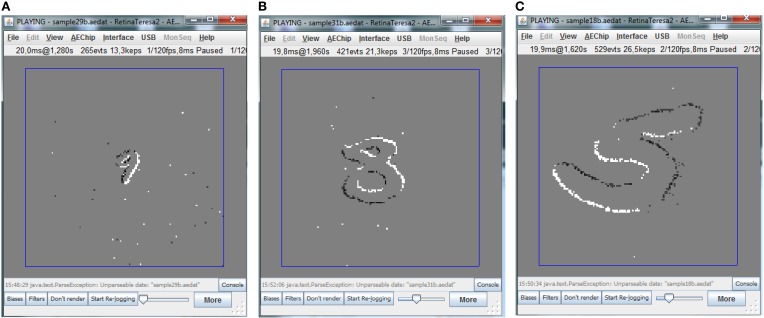
**Example of different moving MNIST digits captured with the DVS camera. (A)** Example of smallest scale digit, **(B)** example of medium scale digit, and **(C)** example of largest scale digit.

For illustrative purposes, Figure 4 shows three jAER snapshots of three MNIST-DVS digits of three different sizes moving over the screen. The moving digits can very easily be stabilized on the screen by simply subtracting the symbol center trajectory (*x*_*c*_(*t*), *y*_*c*_(*t*)) from the recorded events. The Matlab script provided to reshuffle timestamps can also be set to stabilize the moving digits this way. Additionally, it can also be set to remove the event polarity changes.

The original MNIST picture dataset (LeCun et al., 1998) comprises 70,000 pictures, all of the same size. From this set we selected only 10,000, scaling each one up to 3 different sizes on the monitor screen. Our set therefore has only 30,000 jAER event-driven movies. The main reason we decided to record only 1/6 of the original dataset was the huge size of the recordings. The 3-scale MNIST-DVS dataset occupies 10.4 Gbytes. The method for upscaling the original 28 × 28 pixel digits to smooth looking edges on the high definition LCD monitor is based on bilinear interpolation.

### 3.1. Example uses of MNIST-DVS and results

The MNIST-DVS data set was used by Henderson et al. (2015) to explore a new spike based learning rule on a multi-layer spiking network. Ninety percent of the recordings of each character were used for training while the remaining 10% were used for testing. The classification output correctly classified the test input digits 87.41% of the time.

Zhao et al. (2015) used two spiking MNIST datasets to test a newly proposed feed forward spiking neural architecture. The first spiking dataset was artificially generated from the original frame-based MNIST dataset (LeCun et al., 1998) by assigning spikes to active pixels. Random spikes were also added on top to emulate noise corruption. For this dataset, correct recognition of between 47.10 and 91.29% was achieved on the test set depending on the level of injected noise. For the training set correct recognition of between 78.01 and 99.36% was achieved, depending on noise level. For the second MNIST spiking dataset MNIST-DVS was used. Its recognition performance was tested as a function of input event stream length, from a minimum of 100 ms to the full 2 s recordings. For the test set, recognition accuracy ranged from 76.86% for 100 ms inputs to 88.14% for the full 2 s recordings. For the training sets, recognition ranged from 98.86% for 100 ms inputs to 99.13% for the 2 s recordings.

## 4. Statistical analysis of databases

Databases can have hidden statistical biases that may make some algorithms perform better than others simply because they discover such hidden statistics. We analyzed the recorded data to check some of its simple statistical properties.

**Figure 5:**
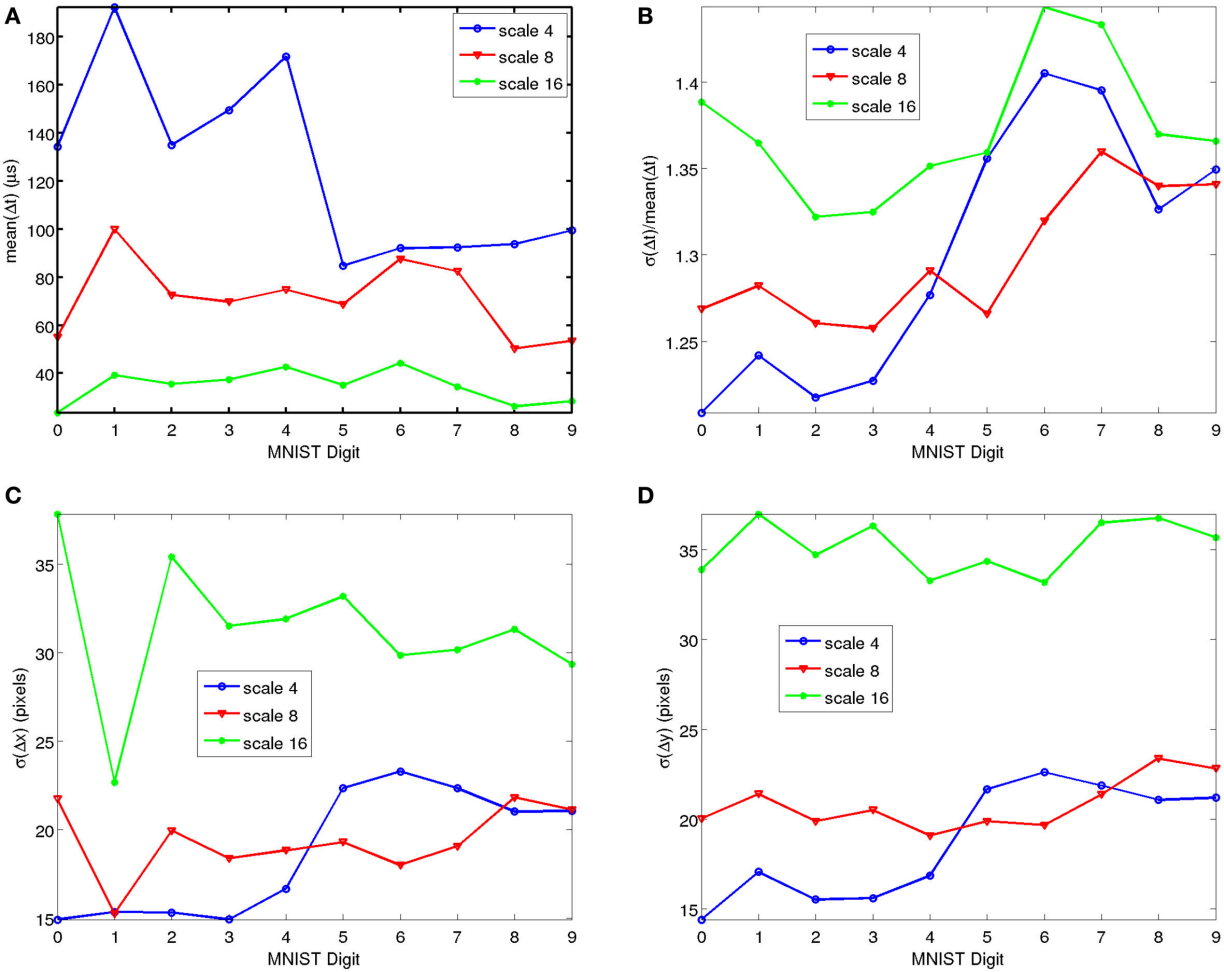
**Simple Statistical Analyses of recorded MNIST-DVS data, as function of MNIST symbol and for the three different scales**. **(A)** Average inter-spike time difference. **(B)** Relative Standard Deviation of inter-spike time differences. **(C)** Standard Deviation of consecutive event *x* coordinate differences. **(D)** Standard Deviation of consecutive event *y* coordinate differences. Matlab scripts to reproduce these figures are included in the Supplementary Material.

For the MNIST-DVS we checked, for each symbol, the average number of events, the mean and standard deviation of the differences in consecutive timestamps Δ*t* of the recorded samples. Figure 5A shows the average $\bar{\Delta t}$ of the inter-spike time differences for each symbol and for the different scales. Since the recording time *T* is always the same for all recording, this average is directly related to the average number of events *N*_*evs*_ per symbol and scale: $\bar{\Delta t}=T∕N_{evs}$. The number of events produced by each symbol is highly related to its edge perimeter. Therefore, it is logical to expect a reasonable variation from symbol to symbol. Figure 5B shows the relative standard deviation of the inter-spike time differences $\sigma \left(\Delta t\right)∕\bar{\Delta t}$, as a function of MNIST symbol and for the different scales. As can be seen, it is fairly stable and symbol independent. We also checked at the *x* and *y* coordinates (see Figures 5C,D) and the polarity bit *p* for simple statistics by computing the mean and standard deviation of *x*, *y*, and *p*, and the mean and standard deviation of consecutive event differences Δ*x*, Δ*y*, and Δ*p*. No bias was revealed either in polarity, the means of the *x* or *y* coordinates, or the means of Δ*x* or Δ*y*. However, for the standard deviations of σ(*x*) ≈ σ(Δ*x*) some weak, symbol-dependent bias was observed, as shown in Figure 5C. In particular digit “1” revealed a smaller spread in the *x* coordinates, although this is quite logical since it is typically much thinner in the *x* direction than the other digits. Consequently, no significant statistical bias has been observed that could favor some algorithms with respect to others.

**Figure 6:**
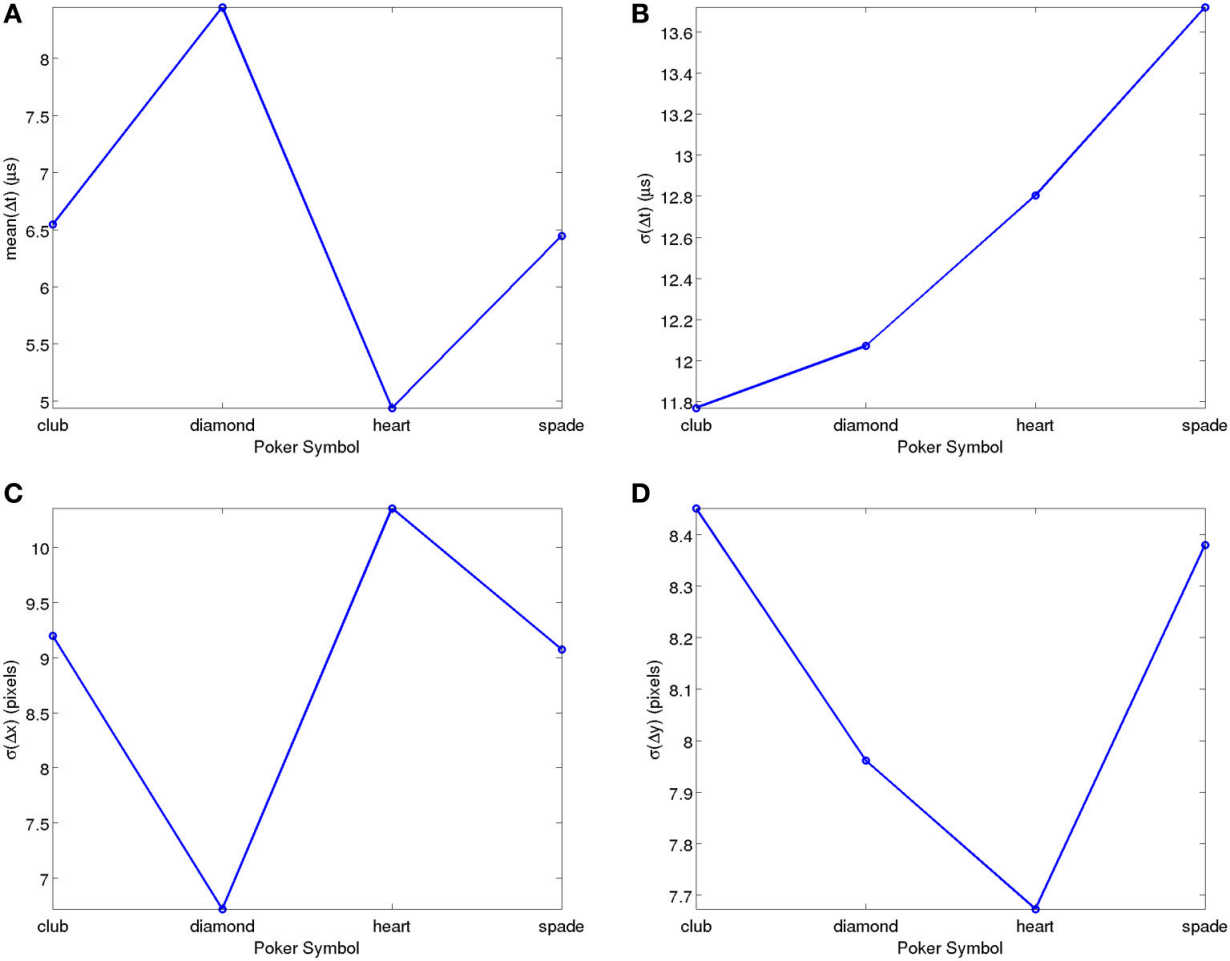
**Simple Statistical Analyses of recorded POKER-DVS data, as function of Poker pip symbol. (A)** Mean of consecutive timestamp differences. **(B)** Standard Deviation of consecutive timestamp differences. **(C)** Standard Deviation of consecutive event *x* coordinate differences. **(D)** Standard Deviation of consecutive event *y* coordinate differences. Matlab scripts to reproduce these figures are included in the Supplementary Material.

Figure 6 shows the result of a similar analysis carried out on the full POKER-DVS database. In this case there is also a symbol-dependent bias in the average inter-spike time differences, most probably also due to perimeter differences. Figure 6A reflects almost a factor 2 spread (between 5 and 9 μs) in $\bar{\Delta t}$. For σ(Δ*t*) the symbol-dependent spread is relatively small. It is interesting to note that, here, it is the absolute standard deviation that has a symbol independent behavior, while in the MNIST-DVS case it was the relative standard deviation. On the other hand, a weak spread can be appreciated in σ(*x*) ≈ σ(Δ*x*) between 6.6 and 10.4 pixels, while for σ(*y*) ≈ σ(Δ*y*) the symbol-dependent spread is even smaller. In summary, no significant statistical bias can be observed for this dataset either.

## 5. Conclusions

This paper presents and discusses the history, background, and some of the hidden details of Poker-DVS and MNIST-DVS, two event-driven datasets developed by our group. Poker-DVS is an extremely high speed dataset (although its timestamps can always be slowed down) which also offers the possibility of simultaneously testing tracking with recognition algorithms. MNIST-DVS is a transformation of the original MNIST digit 28 × 28 pixel pictures into event-driven movies, with a duration of over 2 s each and for three different sizes. Poker-DVS is a very high speed database, and also the moving symbols contain a much smaller number of events than the ones in MNIST-DVS. On the other hand, MNIST-DVS contains 10 different symbols instead of 4, and its input space is 128 × 128 pixels instead of 32 × 32. In any case, both datasets present a good balance between speed and complexity, and could constitute not only good starting points for event-driven vision tests but also inspiration for future benchmarks.

### Conflict of interest statement

The authors declare that the research was conducted in the absence of any commercial or financial relationships that could be construed as a potential conflict of interest.

## Footnotes

^1^In these asynchronous pixel arrays, events are read out through competing arbitration. When there are more than one event request to be read out simultaneously, all except one will be delayed a few tens of nano seconds. However, if pixel requests in the array exceed the maximum rate the arbiter can handle, then the arbiter saturates and event read out collapses.

## References

[B1] Boahen K. (2000). Point-to-point connectivity between neuromorphic chips using address events. IEEE Trans. Circ. Syst. Part II 47, 416–434. 10.1109/82.842110

[B2] Brandli C. Berner R. Yang M. Liu S.-C. Delbrück T. (2014). A 240 × 180 130 dB 3μ*s* latency global shutter spatio-temporal vision sensor. IEEE J. Solid State Circ. 49, 2333–2341. 10.1109/JSSC.2014.2342715

[B3] Camuñas-Mesa L. Zamarreno-Ramos C. Linares-Barranco A. Acosta-Jiménez A. Serrano-Gotarredona T. Linares-Barranco B. (2012). An event-driven multi-kernel convolution processor module for event-driven vision sensors. IEEE J. Solid State Circ. 47, 504–517. 10.1109/JSSC.2011.2167409

[B4] Chen S. Bermak A. (2005). A low power CMOS imager based on time-to-first-spike encoding and fair AER, in IEEE International Symposium on Circuits and Systems, 2005. ISCAS 2005, Vol. 5 (Kobe), 5306–5309.

[B5] Costas-Santos J. Serrano-Gotarredona T. Serrano-Gotarredona R. Linares-Barranco B. (2007). A spatial contrast retina with on-chip calibration for neuromorphic spike-based AER vision systems. IEEE Trans. Circ. Syst. I 54, 1444–1458. 10.1109/TCSI.2007.900179

[B6] Culurciello E. Etienne-Cummings R. Boahen K. (2003). A biomorphic digital image sensor. IEEE J. Solid State Circ. 38, 281–294. 10.1109/JSSC.2002.807412

[B7] Delbrück T. (2006). Available online at: http://sourceforge.net/projects/jaer/

[B8] Fei-Fei L. Fergus R. Peron P. (2007). Learning generative visual models from few training examples: an incremental Bayesian approach tested on 101 object categories. Comput. Vis. Image Underst. 106, 59–70. 10.1016/j.cviu.2005.09.012

[B9] Henderson J. Ting T. Gibson A. Wiles J. (2015). Spike event based learning in neural networks. arXiv. Available online at: http://arxiv.org/abs/1502.05777

[B10] Lagorce X. (2015). HOTS: a Hierarchy of Event-Based Time-Surfaces for Pattern Recognition. Ph.D. Dissertation, Computational Methods for Event Based Signals and Applications, University Pierre et Marie Curie, Paris.

[B11] LeCun Y. Bottou L. Bengio Y. Haffner P. (1998). Gradient-based learning applied to document recognition. Proc. IEEE 86, 2278–2324. 10.1109/5.726791

[B12] Leñero-Bardallo J. Bryn D. Hafliger P. (2014). Bio-inspired asynchronous pixel event tricolor vision sensor. IEEE Trans. Biomed. Circ. Syst. 8, 345–357. 10.1109/TBCAS.2013.2271382 23934671

[B13] Leñero-Bardallo J. Serrano-Gotarredona T. Linares-Barranco B. (2010). A five-decade dynamic-range ambient-light-independent calibrated signed-spatial-contrast AER retina with 0.1-ms latency and optional time-to-first-spike mode. IEEE Trans. Circ. Syst. I 57, 2632–2643. 10.1109/TCSI.2010.2046971

[B14] Leñero-Bardallo J. Serrano-Gotarredona T. Linares-Barranco B. (2011). A 3.6us latency asynchronous frame-free event-driven dynamic-vision-sensor. IEEE J. Solid State Circ. 46, 1443–1455. 10.1109/JSSC.2011.2118490

[B15] Lichtsteiner P. Posch C. Delbrück T. (2008). A 128 × 128 120dB 30mW asynchronous vision sensor that responds to relative intensity change. IEEE J. Solid State Circ. 43, 566–576. 10.1109/JSSC.2007.914337

[B16] Orchard G. Jayawant A. Cohen G. K. Thakor N. (2015a). Converting static image datasets to spiking neuromorphic datasets using saccades. Front. Neurosci. 9:437. 10.3389/fnins.2015.00437 26635513 PMC4644806

[B17] Orchard G. Meyer C. Etienne-Cummings R. Posch C. Thakor N. Benosman R. (2015b). HFirst: a temporal approach to object recognition. IEEE Trans. Pattern Anal. Mach. Intell. 37, 2028–2040. 10.1109/TPAMI.2015.2392947 26353184

[B18] Pérez-Carrasco J. A. Acha B. Serrano C. Camuñas-Mesa L. Serrano-Gotarredona T. Linares-Barranco B. (2010). Fast vision through frameless event-based sensing and convolutional processing: application to texture recognition. IEEE Trans. Neural Netw. 21, 609–620. 10.1109/TNN.2009.2039943 20181543

[B19] Pérez-Carrasco J. A. Zhao B. Serrano C. Acha B. Serrano-Gotarredona T. Chen S. Linares-Barranco B. (2013). Mapping from frame-driven to frame-free event-driven vision systems by low-rate rate coding and coincidence processing. Application to feedforward ConvNets. IEEE Trans. Pattern Anal. Mach. Intell. 35, 2706–2719. 10.1109/TPAMI.2013.71 24051730

[B20] Posch C. Matolin D. Wohlgenannt R. (2011). A QVGA 143 dB dynamic range frame-free PWM image sensor with lossless pixel-level video compression and time-domain CDS. IEEE J. Solid State Circ. 46, 259–275. 10.1109/JSSC.2010.2085952

[B21] Serrano-Gotarredona R. Öster M. Lichtsteiner P. Linares-Barranco A. Paz-Vicente R. Gómez-Rodríguez F. (2009). CAVIAR: a 45k neuron, 5M synapse, 12G connect/s AER hardware sensory-processing-learning-actuating system for high speed visual object recognition and tracking. IEEE Trans. Neural Netw. 20, 1417–1438. 10.1109/TNN.2009.2023653 19635693

[B22] Serrano-Gotarredona T. Linares-Barranco B. (2013a). A 128 × 128 1.5 4 mW asynchronous frame-free dynamic vision sensor using transimpedance preamplifiers. IEEE J. Solid State Circ. 48, 827–838. 10.1109/JSSC.2012.2230553

[B23] Serrano-Gotarredona T. Linares-Barranco B. (2013b). Available online at: http://www2.imse-cnm.csic.es/caviar/MNISTDVS.html

[B24] Serrano-Gotarredona T. Linares-Barranco B. (2015). Available online at: http://www2.imse-cnm.csic.es/caviar/POKERDVS.html

[B25] Van Rullen R. Thorpe S. (2001). Rate coding versus temporal order coding: what the retinal ganglion cells tell the visual cortex. Neural Comput. 13, 1255–1283. 10.1162/08997660152002852 11387046

[B26] Yang M. Liu S.-C. Delbrück T. (2015). A dynamic vision sensor with 1% temporal contrast sensitivity and in-pixel asynchronous delta modulator for event encoding. IEEE J. Solid State Circ. 50, 2149–2160. 10.1109/jssc.2015.2425886

[B27] Zhao B. Ding R. Chen S. Linares-Barranco B. Tang H. (2015). Feedforward categorization on AER motion events using cortex-like features in a spiking neural network. IEEE Trans. Neural Netw. Learn. Syst. 26, 1963–1978. 10.1109/tnnls.2014.2362542 25347889

